# Bromocriptine and cabergoline induce cell death in prolactinoma cells via the ERK/EGR1 and AKT/mTOR pathway respectively

**DOI:** 10.1038/s41419-019-1526-0

**Published:** 2019-04-18

**Authors:** Chao Tang, Ruixin Sun, Guodao Wen, Chunyu Zhong, Jin Yang, Junhao Zhu, Zixiang Cong, Xiaoying Luo, Chiyuan Ma

**Affiliations:** 1 Department of Neurosurgery, Jinling Hospital, School of Medicine, Nanjing University, 305 East Zhongshan Road, 210002 Nanjing, China; 2 State Key Laboratory of Oncogenes and Related Genes, Shanghai Cancer Institute, Renji Hospital, Shanghai Jiaotong University School of Medicine, 200032 Shanghai, China; 3 Tungwah Hospital of Sun Yat-Sen University, Dongguan, China; 4 School of Medicine, Nanjing Medical University, 104 Hanzhong Road, 210002 Nanjing, China

**Keywords:** Endocrinology, Endocrine system and metabolic diseases

## Abstract

The treatment of hyperprolactinemia is based on the use of dopamine agonists, mainly bromocriptine (BRC) and cabergoline (CAB). They reduce tumour size effectively and restore gonadal function. However, there is a difference in drug sensitivity between CAB and BRC in patients with prolactinoma, although the underlying mechanisms are still unknown. Thus, we investigated whether there are differences in tumour sensitivity to CAB and BRC and their possible differential mechanisms in two prolactinoma cell lines. In our study, we found that GH3 cells are more sensitive to BRC and that MMQ cells are more sensitive to CAB. Moreover, BRC and CAB elicited cell death via different pathways; BRC induced prolactinoma cell death mainly through the apoptosis pathway, and CAB induced pituitary prolactinoma cell death mainly via the autophagic cell death pathway. Using gene microarray analysis, we found that BRC induces the apoptosis of prolactinoma cells through the ERK/EGR1 signalling pathway, whereas CAB induces autophagic death by inhibiting the AKT/mTOR signalling pathway. Our study showed the difference in tumour sensitivity and differential mechanisms in BRC- and CAB-treated prolactinoma cells, which provides a theoretical basis for the accurate treatment of prolactinoma.

## Introduction

Prolactinomas are the most common type of pituitary tumour and are responsible for numerous cases of hyperprolactinemia, which can lead to oligomenorrhea, amenorrhea or galactorrhea syndromes in women as well as erectile dysfunction and decreased libido in men^1,2^. Giant prolactinomas, which are fortunately rare clinical events^3^, are defined as unusually large tumours (larger than 4 cm in maximal diameter) with extremely high serum prolactin (PRL) concentrations (above 1000 ng/ml) and obvious mass-effect symptoms, such as headache and visual field defects (VFDs)^4^. Due to their invasive clinical behaviour, giant prolactinomas are particularly difficult to treat^4^. The major objectives of treatment for prolactinomas are to reduce the tumour mass, to relieve the neurological symptoms and to control the excess PRL secretion^5^.

Dopamine agonists, mainly bromocriptine (BRC) and cabergoline (CAB), are the first-line treatment for the majority of patients with idiopathic hyperprolactinemia and prolactinomas, and they effectively suppress prolactin secretion and shrink tumour volume in most patients^6,7^. BRC was the first drug used for the treatment of prolactinoma, and its clinical application has spanned nearly 30 years^7^. Clinical studies have shown that BRC can effectively control serum prolactin levels in 80–90% of microadenomas and 70% of large adenomas and can effectively restore gonadal function in patients and reduce tumour volume^8,9^. CAB is a dopamine agonist widely used clinically for the treatment of pituitary adenomas and Parkinson’s disease. It is the first choice for the treatment of prolactinomas, because it effectively reduces PRL secretion and shrinks tumours in most patients^2,10^. However, studies have shown that there is a specific difference in drug sensitivity between CAB and BRC; in patients with BRC resistance, CAB treatment is used to achieve a good clinical effect^11,12^. In a small number of patients in the clinical setting, the preferred CAB treatment does not normalize serum PRL levels and may fail to shrink the tumour by >50%, even at very high doses; these patients may respond to BRC^13^. This indicates that there is a difference in the tumour sensitivity to CAB and BRC in patients with prolactinoma. Therefore, clarifying the different mechanisms by which CAB and BRC act on prolactinoma appears to be important. In this study, we investigated whether there are differences in the sensitivity of cells to CAB and BRC and evaluated the possible mechanisms by which CAB and BRC induce cell death in different prolactinoma cell lines. These findings elucidate novel mechanisms by which CAB and BRC act, providing a reference for clinical practice.

## Materials and methods

### Cell culture

MMQ cells and GH3 cells (purchased from the Cell Culture Centre, Institute of Basic Medical Sciences, Chinese Academy of Medical Sciences, China) were cultured in Ham’s F10 medium and F12 medium containing 15% horse serum, 2.5% foetal calf serum, and 1% penicillin and streptomycin and were maintained at 37 °C in a 5% CO_2_ atmosphere.

### Animal model

Five-week-old female athymic nude mice were purchased from the SLAC (Shanghai, China). GH3 cells (1 × 10^6^) in PBS were subcutaneously injected into the right side of the back of each nude mouse. The animals were assigned randomly to two groups, and the tumours were allowed to grow to ~50 mm^3^ in size. At this point, BRC (0.5 mg/kg/d) in 100 μl of 0.9% saline was administered daily. Tumour volumes were measured with a Vernier caliper twice a week and calculated as (length × width^2^)/2. All procedures were performed in accordance with the National Institutes of Health Guide for the Care and Use of Laboratory Animals.

### Quantitative assessment of apoptosis

A quantitative assessment of apoptosis was performed with an Annexin-V fluorescein isothiocyanate (FITC) assay kit (Nanjing Keygen Biotech. Co. Ltd., Nanjing, China). Briefly, GH3 and MMQ cells (5 × 10^5^) were plated in six-well plates and serum-starved for 12 h. Then, the indicated amount of paeoniflorin was added to the cells. After 48 h, the cells were collected, washed twice with ice-cold 1 × PBS buffer, suspended in binding buffer, and stained with Annexin V fluoresce FITC and propidium iodide. Cells were analysed by flow cytometry according to the manufacturer’s instructions.

### Cell counting K-8 (CCK8)

Cell viability was measured using the Cell Counting Kit-8 (Dojindo, Kumamoto, Japan). According to the manufacturer’s instructions, log-phase MMQ and GH3 cells were collected and adjusted to 1 × 10^5^ cells/ml. A 100-μL aliquot of the cell suspension was added into each well in a 96-well plate. After serum starvation for 12 h, the cells were treated with fresh medium or various concentrations of BRC or CAB dissolved in medium for 48 h. Ten microliters of the CCK-8 stock solution was added to the media. The optical density (OD) was determined at 450 nm in a Spectra Max M5 (MD, California).

### Western blot analysis

Cells were washed three times with 1 × PBS and lysed in RIPA buffer with a protease inhibitor cocktail and PMSF (200 μg/ml). Equal amounts of protein were separated by sodium dodecyl sulfate polyacrylamide gel electrophoresis and transferred to polyvinylidene difluoride membranes (Millipore, Billerica, USA). The membranes were blocked with 5% non-fat dry milk in PBS containing 0.1% Tween-20 for 1 h at room temperature. Subsequently, the membranes were exposed to primary antibodies diluted to 1:1000 in 5% non-fat dry milk in TBS overnight at 4 °C. After washing the cells and incubating them with horseradish peroxidase-conjugated secondary antibodies for 1 h at room temperature, the immunoreactive complexes were visualized using a chemiluminescent substrate, and the images were acquired on film.

### RNA isolation and RNA interference

Total RNA was extracted using Trizol reagent. One microgram of total RNA was used for first-stand cDNA synthesis according to the manufacturer’s protocol. The sequences of the RT-PCR primers are as follows: Egr1 sense: AGCCTTCGCTCACTCCACTA, antisense: GACTCAACAGGGCAAGCATAC (184 bp); β-actinsense: TGCTATGTTGCCCTAGACTTCG, antisense: GTTGGCATAGAGGTCTTTACGG (240 bp). Gene expression was analysed by RT-qPCR using the standard curve method on an ABI 7500 Real-Time PCR System. The siRNAs were designed and synthesized by Abm (Zhenjiang, China). GH3 and MMQ cells were transfected at ~50% confluence with siRNAs using Lipofectamine RNAi MAX, and the sequences of the successful siRNAs were as follows:siEGR1-676: CCTGACTATCTGTTTCCACAACAACAGGG;siEGR1-825: GGACTTAAAGGCTCTTAATAACACCTACC;siEGR1-1026: GTGTCGAATCTGCATGCGTAATTTCAGTC; and siEGR1-1446: GGTCAGCAACTCCTTCAGCACCTCAACGG.

### Electron microscopy

The cells were fixed with 2% glutaraldehyde for 2 h and then post-fixed in 1% osmium tetroxide for 1 h. Dehydration was induced in increasing concentrations of ethanol followed by dehydration in propylene oxide. When it was incubating in 70% ethanol, the pellet was stained enblock with 1% uranyl acetate. Finally, the pellet was embedded in epon resin. Ultrathin sections were routinely post-stained with uranyl acetate and Reynold’s lead citrate. Electron micrographs were acquired using an electron microscope.

### Microarray

The mRNA expression profiles were analysed using the Affymetrix Gene-Chip array. Total RNA (1 μg), including miRNA from the tissue, was biotin-labelled. The samples were hybridized using a GeneChip® Hybridization Oven to the Affymetrix miRNA microarray according to the protocols provided by the manufacturer. The labelled RNA was heated to 99 °C for 5 min and then to 45 °C for 5 min. RNA-array hybridization was performed with agitation at 60 rotations per minute for 16 h at 48 °C on an Affymetrix® 450 Fluidics Station. The chips were washed and stained using a Genechip Fluidics Station 450 (Affymetrix, Santa Clara, California, United States). The chips were then scanned with an Affymetrix GCS 3000 scanner (Affymetrix, Santa Clara, California, United States). Signal values were computed using the Affymetrix® GeneChip™ Command Console software. The cutoff criterion for differential gene calls was at least two-fold between two compared profiles. The microarray data set was submitted to the GEO repository (GSE101012).

### Immunohistochemical staining

Immunohistochemical staining was performed by incubating tissue sections with mouse EGR1 primary antibody (Santa Cruz Biotechnology, 1:50 in 1% BSA in TBST) overnight at 4 °C with gentle shaking, followed by incubating the sections with goat anti-mouse HRP secondary antibody (1:200 in 1% BSA in TBST) for 1 h at room temperature. The sections were then exposed to DAB substrate (dissolved in Dako substrate buffer) and subjected to a standard dehydration treatment. The staining images were obtained using an Axiovert 200 microscope.

### Statistical analysis

The results are shown as the mean ± SD of four to six experiments, with three repeats of each condition per experiment. Comparisons between various conditions were performed using an unpaired *t-*test. *p*-values less than 0.05 were considered statistically significant. SPSS statistical software version 12.0.1 was used to analyse the data.

## Results

### CAB and BRC decreased the cell viability of rat pituitary adenoma cells

**Fig. 1:**
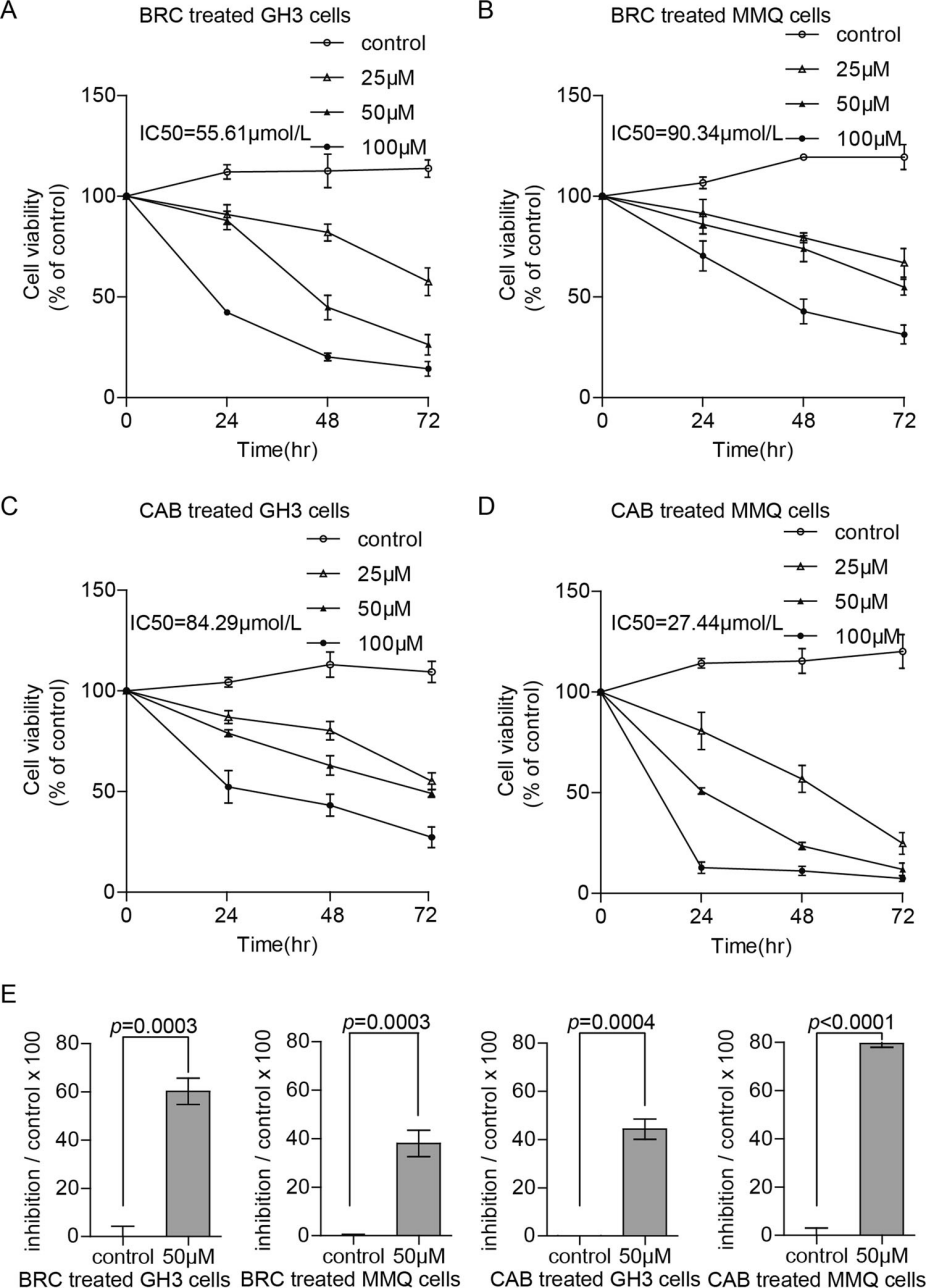
CAB and BRA decreased the viability of GH3 and MMQ cells. **a**–**d** Cell survival was determined by the CCK8 assay. MMQ and GH3 cells were treated with CAB and BRC at concentrations of 0, 25, 50, and 100 μM, respectively, for 24, 48, and 72 h. **e** GH3 and MMQ cells were treated with CAB or BRC at a concentration of 50 μM for 48 h, and cell viability was tested with the CCK8 kit

To test the cell death induced by CAB and BRC, CCK8 assays were used to analyse the viability of GH3 and MMQ cells. GH3 and MMQ cells were treated with CAB or BRC at concentrations of 0, 25, 50, and 100 μM for 24, 48, and 72 h. As shown in Fig. 1a–d, CAB and BRC decreased the viability of GH3 and MMQ cells in both a dose- and time-dependent manner. After 48 h of drug treatment, the IC50 values of CAB and BRC in GH3 cells were 84.29 ± 9.16 μM and 55.61 ± 4.19 μM, respectively, and the IC50 value at which BRC inhibited GH3 cell proliferation was less than that of CAB. The IC50 values of CAB and BRC in MMQ cells were 27.44 ± 10.21 μM and 90.34 ± 7.93 μM, respectively. Treatment with 50 μM BRC for 48 h induced cell death by up to 60% in GH3 cells and 40% in MMQ cells. However, treatment with 50 μM CAB for 48 h induced cell death by 40% in GH3 cells and 80% in MMQ cells (Fig. 1e). These results suggested that BRC had a greater inhibitory effect on GH3 cells than CAB, while CAB had a greater inhibitory effect on MMQ cells than BRC.

### CAB and BRC induced autophagy in rat pituitary adenoma cells

**Fig. 2:**
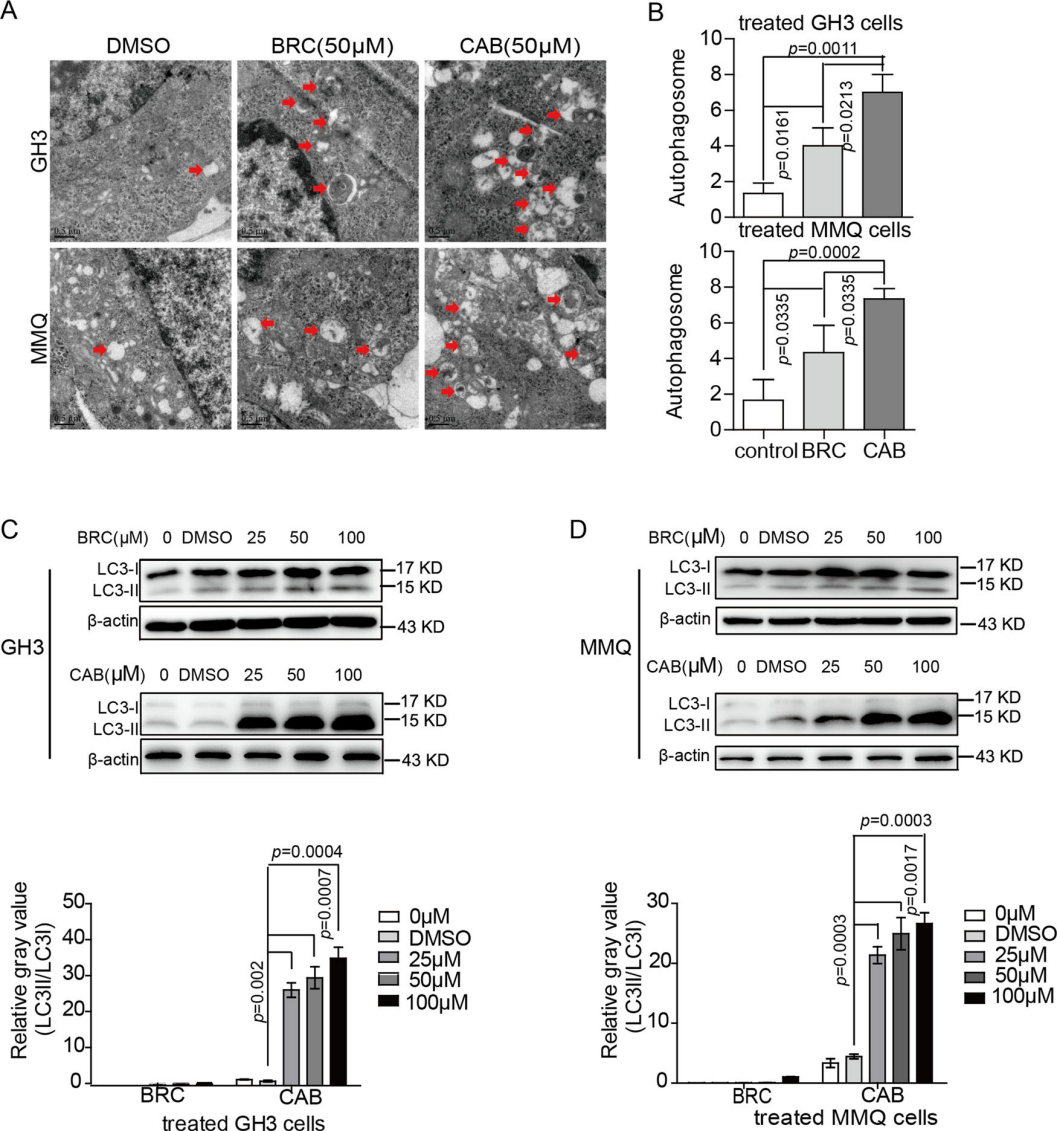
Comparison of CAB- and BRC-induced autophagy in prolactinoma cells. **a**, **b** Electron microscopy images with enlargements. Arrows show autophagic vacuoles in GH3 and MMQ cells treated with BRC (50 μM) or CAB (50 μM) for 48 h. **c**, **d** Western blot analysis of LC3-I and LC3-II in GH3 and MMQ cells with or without BRC (50 μM) or CAB (50 μM) treatment at different concentrations

Prior studies have reported that the over-activation of autophagy can induce autophagic cell death (ACD)^14,15^. To investigate whether CAB and BRC both induce autophagy, GH3 and MMQ cells were treated with BRC or CAB for 48 h, and the autophagosomes were examined by electron microscopy. After 48 h of BRC or CAB exposure, large-scale autophagic vacuoles occurred in the cytoplasm in GH3 and MMQ cells (Fig. 2a). However, compared with the BRC-treated group, the autophagosomes induced by CAB in both cell lines were significantly higher.

During autophagy activation, microtubule-associated protein light chain 3-I (LC3-I) is converted to LC3-II, which is associated with autophagic vesicles and displays classic punctate distribution. This LC3-I to LC3-II conversion is a classic hallmark of autophagy^16^. Western blot results showed that both CAB and BRC could induce the conversion of LC3-I to LC3-II, but the conversion effect of LC3-I to LC3-II in GH3 and MMQ cells induced by CAB was significantly stronger than that induced by BRC (Fig. 2b). c and d), suggesting that CAB strongly induces autophagy in these cells.

### Role of autophagy induced by CAB- and BRC in rat pituitary adenoma cells

**Fig. 3:**
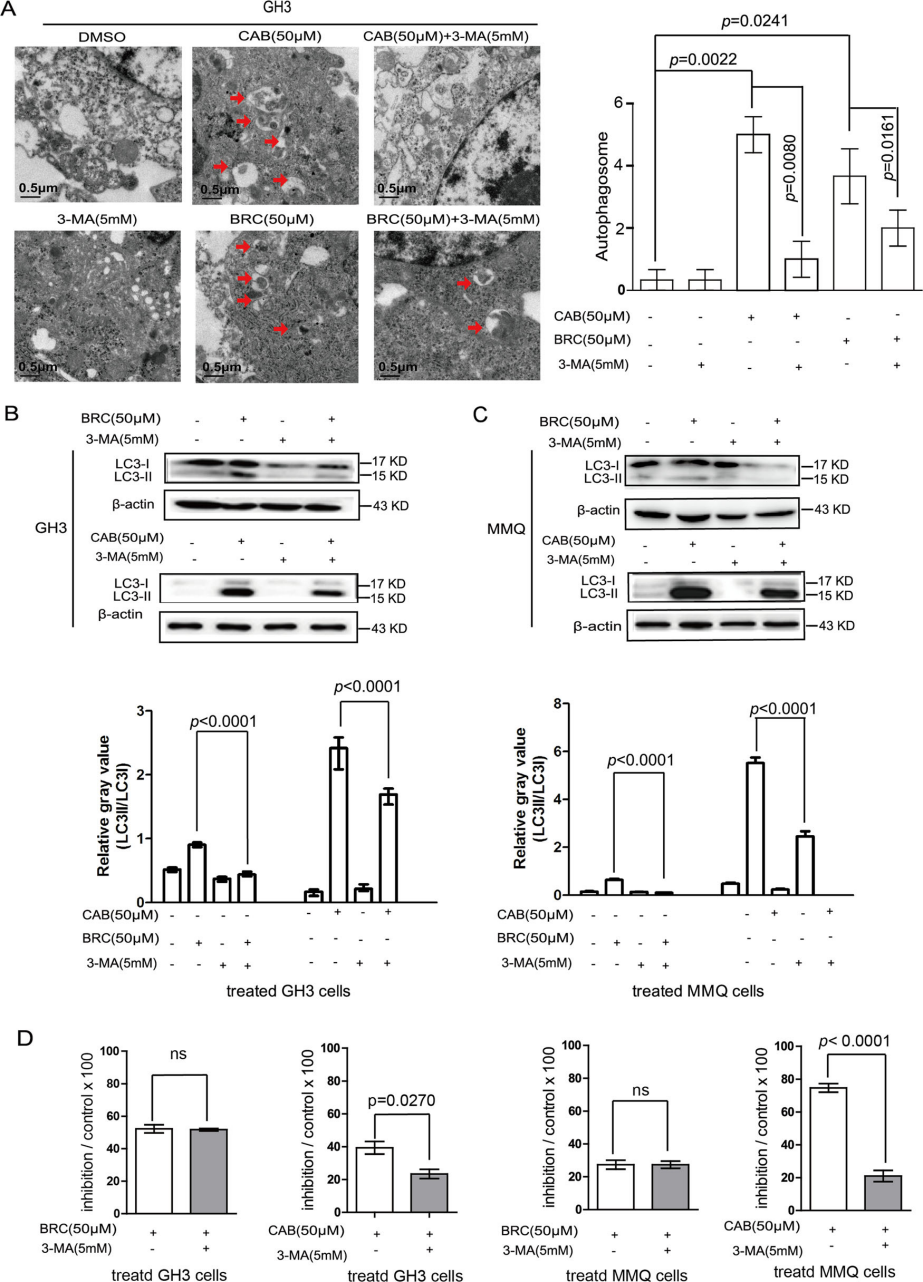
CAB promoted autophagy-induced cell death in prolactinoma cells. **a** Electron micrographs of GH3 and MMQ cells treated with BRC or CAB for 48 h, with or without 3-MA (5 mM). Histogram shows the autolysosome structures from multiple experiments in a total of 50 cells (mean ± SD). **b**, **c** Immunoblot analysis of LC3-I and LC3-II in GH3 and MMQ cells with or without BRC (50 μM) or CAB (50 μM) treatment and with or without 3-MA (5 mM). **d** GH3 and MMQ cells were treated with CAB and BRC at a concentration of 50 μM for 48 h, with or without 3-MA (5 mM), and cell viability was tested with the CCK8 kit

3-Methyladenine (3-MA) can block autophagy through the action of phosphoinositide 3-phosphate kinase (PI3K)^17^. To examine whether increased autophagy was responsible for CAB- and BRC-induced cell death, the viability of GH3 and MMQ cells was tested via the chemical inhibition of autophagy by 3-MA. The autophagosomes induced by CAB or BRC were reduced after treatment with 3-MA in GH3 cells (Fig. 3a). Furthermore, 3-MA effectively inhibited the CAB- and BRC-induced transformation of LC3-I to LC3-II in both cell lines (Fig. 3b, c). On the other hand, 3-MA reversed the CAB-mediated inhibitory effect on both cell types at 48 h but did not significantly reduce the BRC-mediated inhibitory effect on the two cell types at 48 h (Fig. 3d). Together, these results indicate that CAB and BRC both induce autophagy in GH3 and MMQ cells, while although CAB presented a stronger ability to induce autophagy in rat pituitary adenoma cells than BRC. ACD is the predominant mechanism involved in cell death in the pituitary cell lines in response to CAB treatment but is not involved in the response to BRC treatment.

### CAB and BRC both induced apoptosis in rat pituitary adenoma cells

**Fig. 4:**
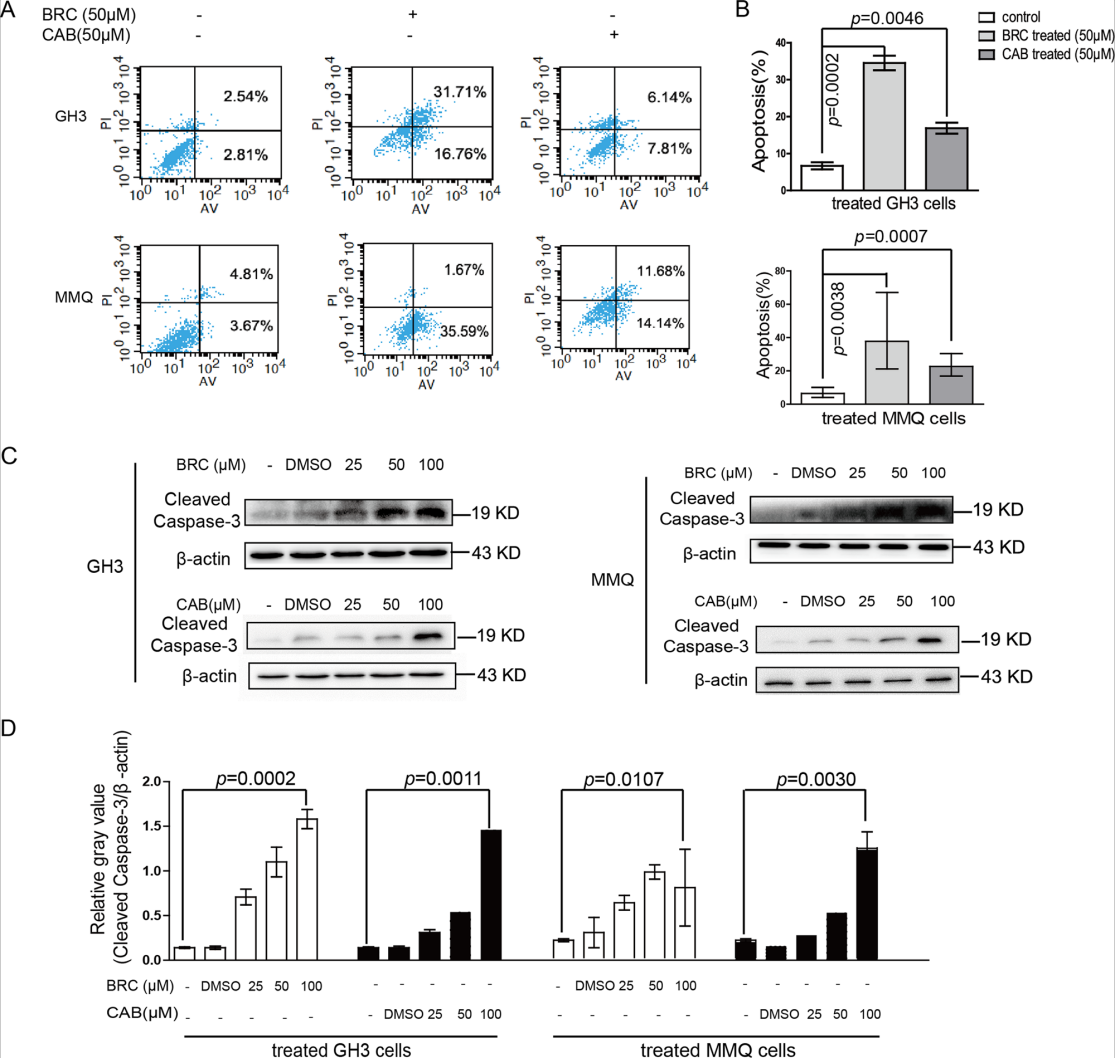
The comparison of CAB- and BRC-induced apoptosis in prolactinoma cells. **a**, **b** MMQ and GH3 cells were treated with CAB (50 μM) or BRC (50 μM) as indicated for the apoptosis assay by Annexin V-FTIC and PI double staining. **c** MMQ and GH3 cells were treated with CAB (50 μM) or BRC (50 μM), and the total proteins were analysed by Western blot using antibodies against cleaved caspase-3 and β-actin

Drug-induced apoptosis is an important process leading to tumour cell death. Thus, apoptosis induction is one of the most important strategies for tumour therapy. Previous studies have demonstrated that CAB and BRC both induce apoptosis in pituitary tumours^18^. However, there have been no studies defining the differences in apoptosis induced by the two drugs. Flow cytometry was used to detect the apoptosis of GH3 and MMQ cells induced by CAB and BRC (Fig. 4a, b). GH3 and MMQ cells were treated with 50 μM CAB or 50 μM BRC for 48 h. The BRC-induced apoptosis rates of both cell lines were significantly greater than that of the control (GH3 cells: control: 6.70 ± 1.64% vs BRC: 38.83 ± 8.97%; MMQ cells: control: 6.50 ± 1.20% vs BRC: 32.11 ± 3.65%). Meanwhile, the CAB-induced cell apoptosis rates of both cell lines were also significantly higher than that of the control (GH3 cells: control: 6.70 ± 1.64% vs CAB: 17.17 ± 2.11%; MMQ cells: control: 6.50 ± 1.20% vs CAB: 22.70 ± 2.63%). Taken together, the BRC-induced cell apoptosis rate was significantly higher than that induced by CAB (Fig. 4a, b).

**Fig. 5:**
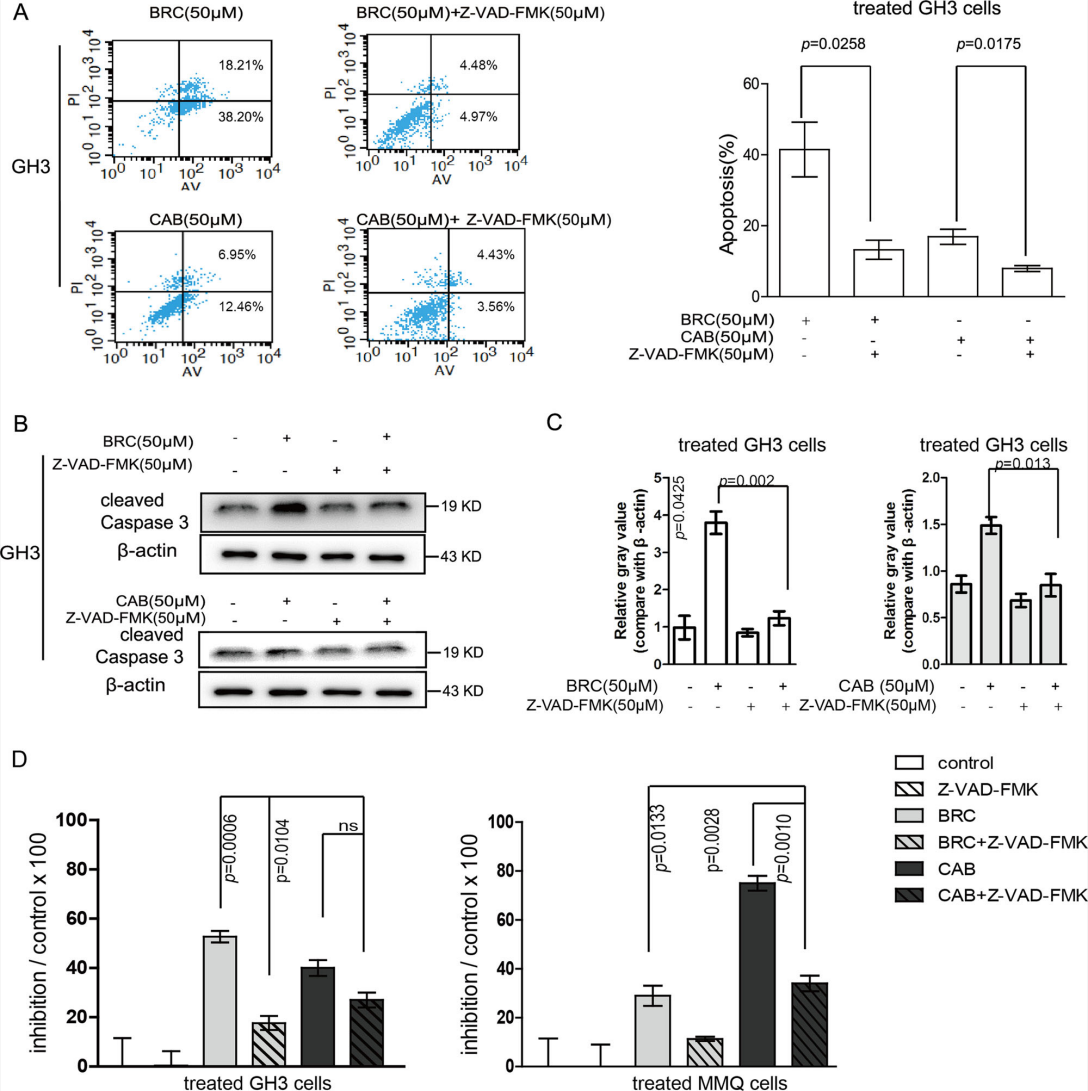
BRC promoted apoptosis-induced cell death in prolactinoma cells. **a** MMQ and GH3 cells were treated with CAB (50 μM) or BRC (50 μM) with or without Z-VAD-FMK (50 μM), as indicated for the apoptosis assay by Annexin V-FTIC and PI double staining. **b**, **c** MMQ and GH3 cells were treated with CAB (50 μM) or BRC (50 μM) with or without Z-VAD-FMK (50 μM), and the total proteins were analysed by Western blot using antibodies against cleaved caspase-3 and β-actin. **d** MMQ and GH3 cells were treated with CAB (50 μM) in the presence or absence of Z-VAD-FMK (50 μM) at 50 μM for 48 h, and cell viability was determined by CCK8

The caspase protein family plays very important roles in drug-induced apoptosis. Among them, caspase-3 is a key molecule that is involved in drug-induced apoptosis. In the early stage of apoptosis, caspase-3 is transformed into activated cleaved caspase-3, which ultimately induces apoptosis^18^. Both CAB and BRC promoted the expression of cleaved caspase-3 in both cell lines. However, the BRC-induced cleaved caspase-3 expression level was significantly higher than that induced by CAB in GH3 and MMQ cells (Figs. 4c and 5d).

Z-VAD-FMK is a cell-permeable caspase inhibitor that can irreversibly block apoptosis^19^. Flow cytometry was used to detect the inhibitory effect of the apoptosis inhibitor Z-VAD-FMK on drug-induced apoptosis (Fig. 5a). Western blotting was used to detect the inhibitory effect of Z-VAD-FMK combined with CAB or BRC in GH3 and MMQ cells. In our study, Z-VAD-FMK significantly inhibited the CAB- or BRC-induced apoptosis of GH3 cells and the expression of cleaved caspase-3 (Fig. 5b, c). In addition, Z-VAD-FMK significantly reduced the inhibition ratios of GH3 and MMQ cells, although with CAB or BRC treatment (Fig. 5d). Together, these results indicated that CAB and BRC can induce apoptosis in GH3 and MMQ cells, although BRC presented a stronger ability to induce apoptosis in GH3 cells than CAB.

### Differential mRNA and pathways participating in BRC- and CAB-induced cell death

**Fig. 6:**
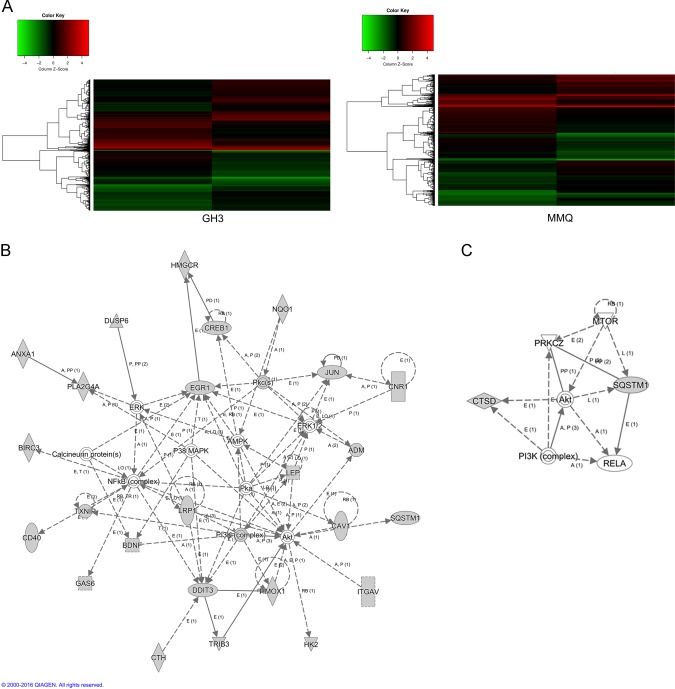
Differential mRNA levels and pathways participating in BRC- and CAB-induced cell death. **a** Differential mRNA expression in GH3 cells treated with or without BRC (50 μM). Differential mRNA expression in MMQ cells treated with or without CAB (50 μM). **b** Pathway enrichment analysis of BRC-treated GH3 cells. **c** Pathway enrichment analysis of CAB-treated MMQ cells

To identify the mRNA and biological pathways associated with CAB- or BRC-treated prolactinoma cells. GH3 cells were treated with BRC for 48 h, and MMQ cells were treated with CAB for 48 h. Figure 6a shows the heatmaps of the microarray data (GeneChip® Rat Genome 230 2.0 Array) describing the mRNA expression profiles after prolactinoma cells were treated with BRC or CAB for 48 h. Upregulated (red spots) and downregulated (green spots) mRNAs showed significantly different expression levels between the control group and drug-treated group (Table 1 and Table 2). Then, the mRNA-target interactions and functional associations were analysed using network-based visual analysis (Fig. 6b). According to the results of the network analysis, the ERK pathway was predicted to be associated with BRC-induced apoptosis in GH3 cells, while the AKT/mTOR pathway was predicted to be associated with CAB-induced autophagy in MMQ cells (Fig. 6b).

**Table 1:** Differential gene expression in GH3 cells treated with BRC compared to untreated cells

Fold change	Log fold change	Regulation	Gene symbol
4.933581	2.3026352	Up	LGALS9
4.930829	2.3018303	Up	EGR1
4.3389397	2.1173425	Up	BLNK
3.6815016	1.8802943	Up	ASTN1
3.6199	1.8559499	Up	KCNMA1
3.4291174	1.7778373	Up	GAA
3.423807	1.7756014	Up	LGALS3
3.358247	1.7477083	Up	HEXB
3.1464825	1.6537399	Up	RORC
2.9402692	1.5559483	Up	DPP7

**Table 2:** Differential gene expression in MMQ cells treated with CAB compared to untreated cells

Fold change	Log fold change	Regulation	Gene symbol
13.250016	3.7279222	Up	CCL2
13.07152	3.708355	Up	RSAD2
10.597276	3.4056215	Up	PCSK9
9.717957	3.280653	Up	GPNMB
7.2340307	2.8547997	Up	ACSS2
7.105637	2.828964	Up	AP1S2
7.084643	2.824695	Up	DDIT3
6.9898877	2.8052692	Up	LGALS3
6.5683384	2.7155285	Up	MVD
6.1448593	2.61938	Up	CCL7
2.8404617	1.5061255	Up	SQSTM1
−10.145994	−3.3428383	Down	SHCBP1
−9.712309	−3.2798142	Down	ATP6V1G3
−8.005065	−3.0009131	Down	CDCA3
−7.996392	−2.999349	Down	TTK
−6.270357	−2.6485476	Down	ASPM

### BRC activates ERK1/2 and upregulates the expression of EGR1 in prolactinoma cells

**Fig. 7:**
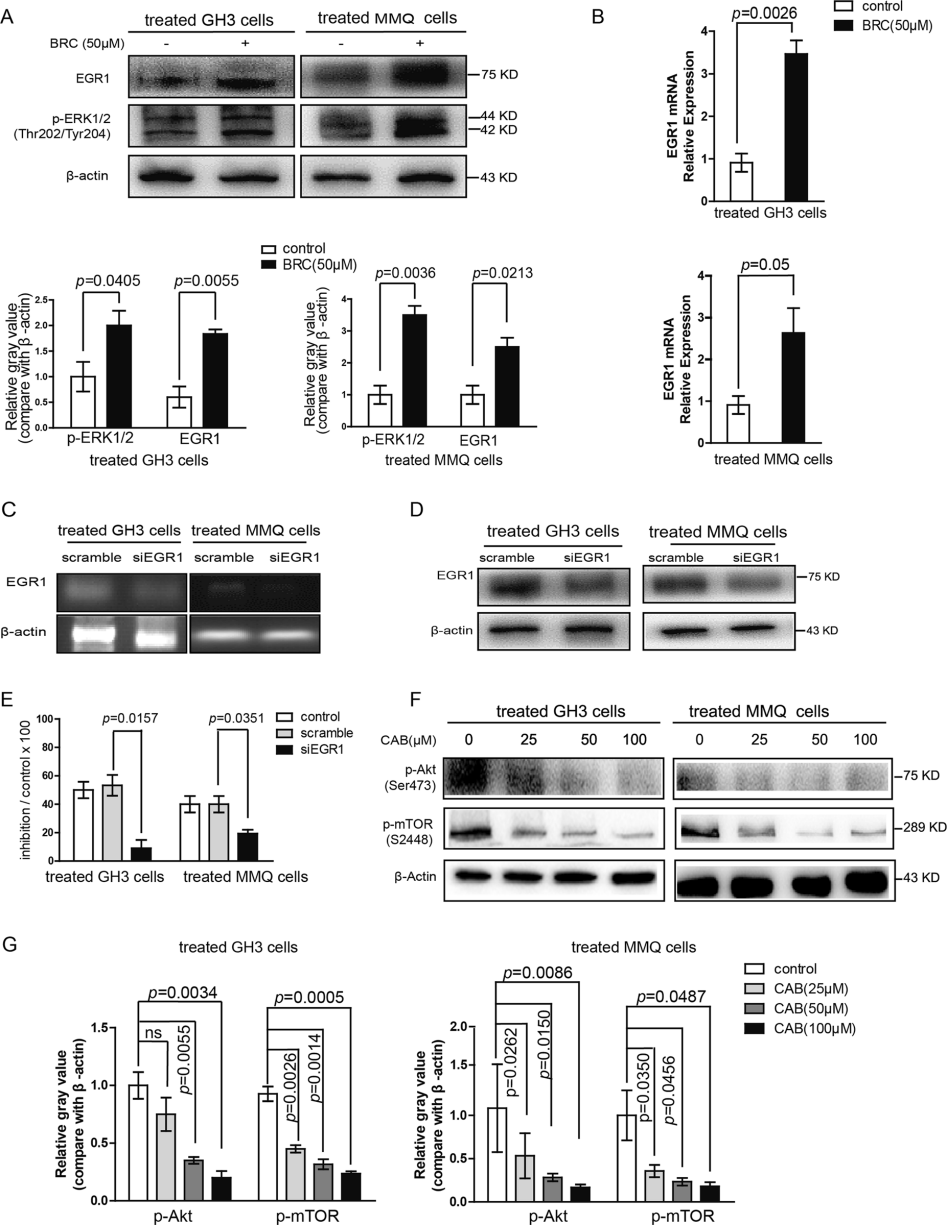
BRC activates ERK1/2 and upregulates the expression of EGR1 in prolactinoma cells. **a** Immunoblot analysis of EGR1 and p-ERK1/2 in GH3 and MMQ cells treated with DMSO or BRC (50 μM) for 48 h. **b** RT-qPCR analysis of the mRNA expression of EGR1 in GH3 and MMQ cells treated with BRC (50 μM) for 48 h. **c**, **d** Immunoblot analysis and RT-PCR of EGR1 expression after GH3 and MMQ cells were transfected with the control (Ctrl) or the siRNA against EGR1 for three days. **e** Cell viability of MMQ and GH3 cells treated with the control (Ctrl) or with the siRNA against EGR1. **f**, **g** GH3 and MMQ cells were treated with CAB at concentrations of 25, 50, and 100 μM, followed by immunoblot analysis for p-Akt, p-mTOR and β-actin

Early growth response protein 1 (EGR1) is one of the classic zinc finger transcription factors. Activation of the ERK pathway could increase EGR1 expression. As shown in Fig. 7a, b, EGR1 expression was increased, and the ERK pathway was significantly activated in GH3 and MMQ cells treated with BRC. After siRNA transfection was used to downregulate EGR-1 (Fig. 7c, d), the inhibitory effect of BRC on the proliferation of rat pituitary adenoma cells was weakened (Fig. 7e). Thus, BRC induces apoptosis in rat pituitary adenoma cells through the ERK/EGR1 pathway.

### Inactivation of AKT/mTOR induced by CAB in pituitary adenoma cells

Next, we identified the effect of CAB on the AKT/mTOR pathway. mTOR is a highly conserved serine/threonine protein kinase. Previous studies have found that AKT could activate the mTOR pathway^20^. Here, we detected the expression levels of p-AKT and p-mTOR in GH3 and MMQ cells treated with CAB for 48 h by western blotting. We found that the phosphorylation levels of AKT and mTOR were significantly lower in the CAB-treated group than in the control group (Fig. 7f, g). These results showed that CAB can suppress the activation of the AKT/mTOR signalling pathway to induce autophagy in prolactinoma cells.

### Subcutaneous xenograft model and drug therapy in nude mice

**Fig. 8:**
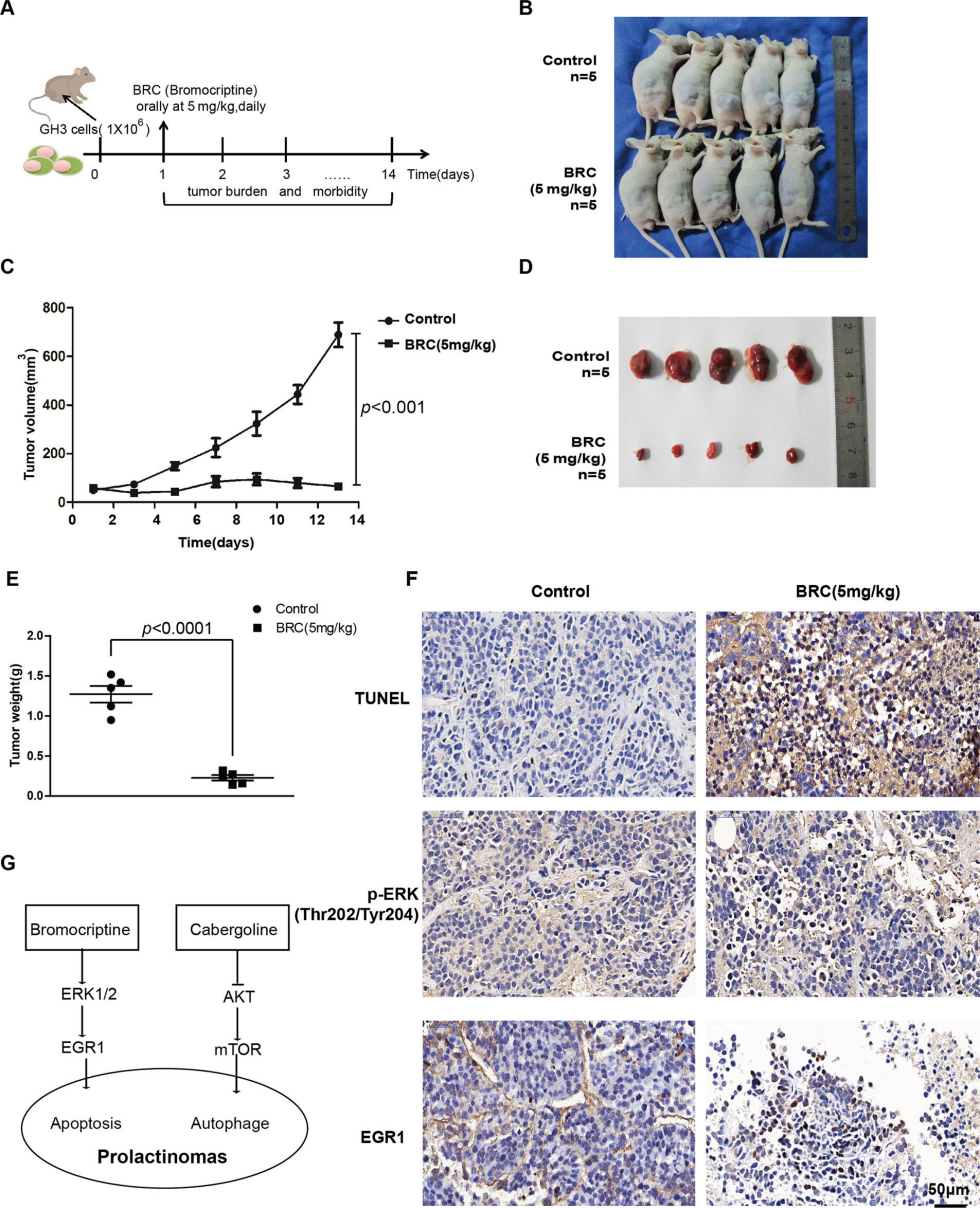
Subcutaneous xenograft model and drug therapy in nude mice. **a** Schematic representation of the experimental procedure employed to study tumours in nude mice. Tumour formation was observed, and tumourigenic nude mice were randomly divided into a control group and a BRC treatment group (*n* = 5). **b** Representative images of xenograft tumours in nude mice. **c** The tumour volume on the nude mouse. **d** Representative images of the tumour samples from each group. **e** The tumour weight of each group. **f** The TUNEL staining and immunohistochemical analyses of EGR1 and p-ERK in tumour samples of each group. **g** The proposed mechanism of BRC- and CAB-induced cell death in prolactinoma cells

Finally, we investigated whether BRC suppressed tumour growth in a GH3 xenograft model. One week after the subcutaneous injection of GH3 cells in nude mice, tumour formation was observed, and tumourigenic nude mice were randomly divided into two groups of 5 mice each (Fig. 8a). We found that the tumour sizes in the BRC treatment group were significantly smaller than those in the control group (65.58 ± 17.85 mm^3^ vs 689 ± 113.15 mm^3^, Fig. 8b–d). The tumour weight was 0.28 ± 0.03 g vs 1.29 ± 0.13 g in the BRC treatment group and control group, respectively. Consistent with these findings, apoptosis was detected by TUNEL staining in the subcutaneously transplanted tumours, and the expression levels of EGR1 and p-ERK1/2 in both groups were detected by immunohistochemistry (Fig. 8f). We found that the positive rate of tumour cell TUNEL staining in the BRC-treated group was significantly higher than that in the control group and that the EGR1 and p-ERK1/2 expression levels were also significantly higher in the BRC treatment group than in the control group (Fig. 8f). These results suggest that BRC suppresses tumour growth by inducing EGR1 in vivo.

## Discussion

In the present study, we investigated the mechanisms underlying the of difference in efficacy between BRC and CAB in patients with prolactinomas using the rat prolactinoma cell lines GH3 and MMQ. For the first time, it was revealed that GH3 cells have a greater sensitivity to BRC than MMQ cells, while MMQ cells have a greater sensitivity to CAB than GH3 cells. Moreover, this difference in sensitivity is likely due to the different pathways of cell death induced by BRC and CAB.

In recent decades, according to morphology, enzymology, immunology and functional characteristics, cell death has mainly been divided into programmed cell death (PCD) and necrosis^14^. PCD is a distinct biochemical and genetic pathway and has been investigated in various cancer cells; its morphologic manifestation of apoptosis is an inherent, controlled cellular death programme^21,22^. Another cell death model involves autophagy, which is also called PCD II to distinguish it from apoptosis or PCD I^23,24^. Apoptosis induction is one of the most important strategies for tumour therapy. Autophagy is a process in which a portion of the cytoplasmic proteins and organelles are engulfed and degraded/digested, which favours cell survival. However, when autophagy is overactivated and too much of the cell contents are lost, cell death is induced. It has been reported that CAB converts autophagy into cell death by the concomitant induction of autophagy and inhibition of autophagic flux^15,16^. BRC was reported to inhibit PRL secretion to shrink the tumour volume and induce apoptosis in GH3 cells^17^. However, it remains unknown whether the other mechanism may also be involved in CAB- and BRC-mediated tumour shrinkage and serum PRL level decrease in prolactinomas.

Autophagy and apoptosis are two main causes of drug-induced cell death. Recently, autophagy-dependent cell death induced by CAB in prolactinoma cells has been reported^25,26^, but there are no reports about BRC-induced cell autophagy. Our data demonstrate that while both BRC and CAB can induce autophagy in GH3 and MMQ cells, CAB-induced autophagy is significantly stronger than BRC-induced autophagy in rat prolactinoma cells. We then inhibited autophagy in the two cells using 3-MA, and the cell viability in the BRC treatment group did not change significantly; however, the cell viability significantly increased with CAB treatment. These results suggested that BRC-induced cell autophagy has little effect on cell viability, while CAB induces cell death mainly through autophagy. However, we could not identify the role of autophagy in BRC-induced cell death.

Next, we focused on the different effects of BRC and CAB on apoptosis in prolactinoma cells. To the best of our knowledge, there has been no comparative study performed investigating the induction of apoptosis by BRC and CAB in prolactinomas cells. We detected the effects of CAB and BRC on apoptosis in GH3 and MMQ cells. Annexin-V staining and the activation of caspase-3, which are features of typical apoptosis, showed the occurrence of apoptosis in GH3 cells and MMQ cells treated with CAB or BRC. However, we found that the apoptosis rates in GH3 and MMQ cells were significantly higher in BRC-treated cells than in the controls. The relative expression of cleaved caspase-3 in cells treated with BRC was also significantly higher than that in CAB-treated cells. After treatment of GH3 cells with Z-VAD-FMK, a pan-caspase inhibitor, we found that the effects of BRC and CAB on apoptosis were significantly inhibited. However, the cell viability of the BRC-treated group was significantly higher than that of the CAB-treated group, suggesting that the apoptosis of prolactinomas cells plays a more important role in BRC-induced cell death than in CAB-induced cell death. By comparing autophagy and apoptosis in GH3 and MMQ cells treated with BRC or CAB, we found that BRC mainly induces the apoptotic effect of prolactinomas cells, while CAB mainly induces autophagy in prolactinomas cells. Taken together, there is a difference in BRC- and CAB-induced cell death in rat pituitary adenoma cells.

Due to the different mechanisms of BRC- and CAB-induced cell death, we used a microarray to define the differences in relative gene expression and signalling pathways between BRC and CAB in pituitary adenoma cells. We analysed the differential gene expression and interaction network in CAB-treated MMQ cells and BRC-treated GH3 cells. We found that the function of BRC was associated with EGR1 expression and the ERK signalling pathway, whereas the function of CAB was related to the activation of the AKT/mTOR pathway, which might explain the difference in BRC- and CAB-induced cell death.

EGR1 is an important transcriptional regulatory factor that regulates cell proliferation, differentiation and cell survival and plays an important role in the processes of tumour growth and differentiation^27^. Studies have shown that a high expression level of EGR1 can significantly inhibit the proliferation of breast cancer, non-small cell lung cancer, and oesophageal cancer^28–30^. The activation of ERK1/2 can upregulate the expression level of EGR1^31,32^. We analysed the levels of ERK1/2 phosphorylation and the EGR1 protein in BRC-treated GH3 and MMQ cells and found that the levels of p-ERK and EGR1 were significantly increased compared to those in the controls. The immunohistochemistry of the subcutaneous xenograft model showed the same result. We then downregulated the expression of EGR1 by siRNA transfection and found that the cell viability of GH3 and MMQ cells was significantly increased after BRC treatment. These results suggested that BRC induces apoptosis through the ERK/EGR1 signalling pathway.

The mTOR pathway can significantly inhibit autophagy^33^. Therefore, to verify whether CAB induces autophagy by inhibiting the mTOR signalling pathway, we detected the expression levels of p-AKT and p-mTOR. We found that CAB can significantly inhibit the activation of p-AKT and p-mTOR. As we have found that CAB can induce autophagy in GH3 and MMQ cells, the AKT/mTOR signalling pathway might play an important role in CAB-induced autophagy.

In summary, we have reached the following conclusions. First, GH3 cells are more sensitive to BRC than MMQ cells, and MMQ cells are more sensitive to CAB than GH3 cells. Second, BRC mainly induces apoptosis, while CAB induces autophagy in prolactinoma cells. Third, BRC induces apoptosis through the ERK/EGR1 signalling pathway, while CAB induces autophagy by inhibiting the AKT/mTOR signalling pathway in prolactinoma cells (Fig. 8g). Our study was the first to demonstrate the differences in drug sensitivities BRC and CAB and the underlying mechanisms in prolactinoma cells to provide a novel therapeutic strategy for the accurate treatment of prolactinoma and other tumours.
